# Practice guidelines for intramuscular injection in mental health: A delphi method

**DOI:** 10.1192/j.eurpsy.2021.1909

**Published:** 2021-08-13

**Authors:** G. Legrand, C. Guiguet-Auclair, S. Boisgard, O. Traore, J.-P. Lanquetin, H. Viennet, N. Morin, Z. Cardinaud, A. Debost-Legrand, L. Bernard

**Affiliations:** 1 Centre Hospitalier Sainte-marie De Clermont-ferrand, Association Hospitalière Sainte-Marie, Clermont-Ferrand, France; 2 Chu Clermont-ferrand, Cnrs, Sigma Clermont, Institut Pascal, Université Clermont Auvergne, Clermont-Ferrand, France; 3 Chu Clermont-ferrand, Université Clermont Auvergne, CNRS, SIGMA Clermont, ICCF, Clermont-Ferrand, France; 4 Service D’hygiène Hospitalière, CHU de Clermont-Ferrand, Clermont-Ferrand, France; 5 Groupe De Recherche En Soins Infirmiers, CH de Saint-Cyr-au-Mont-d’Or, Saint-Cyr-au-Mont-d’Or, France; 6 Institut De Formation En Soins Infirmiers, CHU de Clermont-Ferrand, Clermont-Ferrand, France

**Keywords:** Delphi method, Intramuscular injections, Nurse practices, Mental health nursing

## Abstract

**Introduction:**

Intramuscular injections (IMI) remain a frequent practice in mental health. The available guidelines for IMI in mental health only focus on the technical side of the practices. Moreover, no recent update has been performed to improve practice of IMI in mental health

**Objectives:**

To assess a formalized consensus agreement regarding the best practice concerning IMI in mental health and to develop practice guidelines.

**Methods:**

A two-round Delphi method was used. The scientific committee consisted in one psychiatrist, one orthopaedic surgeon, one infection control practitioner, one hospital pharmacist, one mental health nurse, one nurse exploring care relationship and one nurse educator. From literature review, each expert proposed specific recommendations. The panel experts were asked to rate the appropriateness and the applicability in current practice of each recommendation on a 9-point Likert scale. Panel members were recruited in five mental health institutions. The first round questionnaire was emailed to each respondent on February 2020 and the second one on June. Propositions were considered appropriate and applicable in current practice if the median was >=7. Agreement among experts were judged by the statistical measure of the Interpercentile Range

**Results:**

From the first round, 46 recommendations were retained by 49 nurses. 27 propositions were retained after this second round by 32 nurses. The scientific committee added 12 other recommendations because of their importance in the literature and clinical practice.

**Conclusions:**

This study provides consensus-based recommendations on IMI in mental health. Nursing staff need to be educated about the new guidelines from both the theoretical and clinical perspectives

**Disclosure:**

No significant relationships.

